# Permeability Enhancers Dramatically Increase Zanamivir Absolute Bioavailability in Rats: Implications for an Orally Bioavailable Influenza Treatment

**DOI:** 10.1371/journal.pone.0061853

**Published:** 2013-04-17

**Authors:** Eric H. Holmes, Harikrishna Devalapally, Libin Li, Michael L. Perdue, Gary K. Ostrander

**Affiliations:** 1 Department of Cell and Molecular Biology, John A. Burns School of Medicine, Honolulu, Hawaii, United States of America; 2 Absorption Systems, LP, Exton, Pennsylvania, United States of America; 3 FluSolutions Consulting, LLC, Hilo, Hawaii, United States of America; “Mario Negri” Institute for Pharmacological Research, Italy

## Abstract

We have demonstrated that simple formulations composed of the parent drug in combination with generally regarded as safe (GRAS) permeability enhancers are capable of dramatically increasing the absolute bioavailability of zanamivir. This has the advantage of not requiring modification of the drug structure to promote absorption, thus reducing the regulatory challenges involved in conversion of an inhaled to oral route of administration of an approved drug. Absolute bioavailability increases of up to 24-fold were observed when Capmul MCM L8 (composed of mono- and diglycerides of caprylic/capric acids in glycerol) was mixed with 1.5 mg of zanamivir and administered intraduodenally to rats. Rapid uptake (t_max_ of 5 min) and a C_max_ of over 7200 ng/mL was achieved. Variation of the drug load or amount of enhancer demonstrated a generally linear variation in absorption, indicating an ability to optimize a formulation for a desired outcome such as a targeted C_max_ for enzyme saturation. No absorption enhancement was observed when the enhancer was given 2 hr prior to drug administration, indicating, in combination with the observed t_max_, that absorption enhancement is temporary. This property is significant and aligns well with therapeutic applications to limit undesirable drug-drug interactions, potentially due to the presence of other poorly absorbed polar drugs. These results suggest that optimal human oral dosage forms of zanamivir should be enteric-coated gelcaps or softgels for intraduodenal release. There continues to be a strong need and market for multiple neuraminidase inhibitors for influenza treatment. Creation of orally available formulations of inhibitor drugs that are currently administered intravenously or by inhalation would provide a significant improvement in treatment of influenza. The very simple GRAS formulation components and anticipated dosage forms would require low manufacturing costs and yield enhanced convenience. These results are being utilized to design prototype dosage forms for initial human pharmacokinetic studies.

## Introduction

Seasonal influenza outbreaks generally cause between 3 and 5 million annual cases and lead to 250,000 to 500,000 deaths world-wide [1]. In cases of pandemic influenza, resulting in widespread and sustained transmission of the disease, hundreds of millions can be infected, with a corresponding increase in deaths [2]. Particularly worrisome are the appearances of a novel hemagglutinin subtypes to which there is no population immunity or the possibility of initiation of human-to-human transmission from a highly lethal animal flu strain. Vaccination may certainly be an effective means for prevention, however, vaccines are strain-specific, and vaccines formulated for one flu season are often ineffective in subsequent seasons due to the rapid evolution of the virus, giving rise to new circulating strains. Protection with vaccines, while proven, can also have variable effectiveness in different settings and age groups. Alternatively, antiviral drugs such as neuraminidase inhibitors are less sensitive to differing strains and can be used to treat influenza in all age groups by reducing the severity of symptoms and shortening the duration of the illness [3].

Influenza A and B virus particles are composed of multiple proteins encasing approximately seven or eight pieces of negative sense viral RNA [4]. The two major virus particle surface glycoproteins are hemagglutinin and neuraminidase (3). Hemagglutinin has lectin activity and binds to terminal α2→6 and/or α2→3 sialic acid residues on N- and O-linked surface proteins of the host cell, mediating cell infection and accumulation of replicated viruses. Neuraminidase subsequently cleaves these sialic acid residues, allowing release of replicated virus from the host cell. Failure of neuraminidase cleavage of the newly replicated virus particles prevents their release and stops subsequent host cell infection and ultimately further viral replication [3], [4].

Studies demonstrate that the substrate binding pocket of influenza virus neuraminidase is conserved among strains, and its X-ray structure has been determined [5]. Based on this information, rational drug design has given rise to a class of viral neuraminidase-specific inhibitors that have been shown to bind with high affinity and have therapeutic utility in treatment of influenza [6]–[8]. Consequently, inhibition of viral neuraminidase has become a major therapeutic approach in the treatment of influenza, with several approved drugs including the neuraminidase-specific inhibitors oseltamivir, zanamivir, peramivir and laninamivir. Of these inhibitors, two have been approved by the Food & Drug Administration (FDA) for treatment of influenza, Tamiflu® (oseltamivir), marketed by Roche, which is orally delivered, and Relenza® (zanamivir), marketed by GlaxoSmithKline (GSK), which is inhaled. In Japan, two additional drugs, Rapiacta® (peramivir-IV) and Inavir® (laninamivir-inhaled), are approved.

Inhibitors such as oseltamivir carboxylate, zanamivir, and peramivir were developed through structure-based drug design and are generally transition-state analogs of sialic acid having high affinity and specificity for multiple subtypes of viral neuraminidase [9]–[13]. These inhibitors are generally 5- or 6-member ring structures with multiple side chains, the most significant being a carboxylic acid group and a basic group in the form of either a primary amine or a guanidino group. These compounds are highly polar, not metabolized *in vivo*, and, although they are effective viral neuraminidase inhibitors, the high polarity and lack of transporter protein binding generally results in under 2% oral bioavailability [3], [14]. In such instances, administration routes such as inhalation or intravenous have either been used or are proposed. Other approaches are also being explored to enhance permeability and more conveniently administer these drugs (e.g., via oral dosing). Owing to recent circulation of influenza strains that have become resistant to oseltamivir, it is generally agreed that oral availability of an alternative neuraminidase inhibitor would provide a useful tool in the influenza antivirals arsenal. This can include multi-binding-site inhibitors computationally designed to target simultaneously several adjacent binding sites of such strains as H5N1 or the oseltamivir-resistant H274Y variant [15]. Interestingly, those compounds determined to have the most favorable ligand efficiency index for multi-site binding to neuraminidase are also highly polar and contain guanidino groups suggesting poor oral bioavailability and potential suitability for the approach described herein [15].

One exception to the otherwise low oral bioavailability of viral neuraminidase inhibitors has been achieved with a prodrug wherein the carboxyl group of oseltamivir carboxylate has been modified to form an ethyl ester, thus reducing the polarity of the molecule. The oral bioavailability of oseltamivir has been reported to be at least 35% in contrast to the 2% oral bioavailability of its oseltamivir carboxylate precursor [16], [17]. Analogous modifications of either zanamivir or peramivir have not improved oral bioavailability, as the much higher polarity of their guanidino group compared to the primary amine of oseltamivir still prevents substantial oral absorption. As a result, to date, neuraminidase inhibitors that contain a guanidino group (such as zanamivir and peramivir) have been resistant to efficacious oral formulation development. A new prodrug strategy has been employed by Amidon's group [18] using amino acid derivatives of zanamivir to target the intestinal membrane transporter hPepT1 to increase drug absorption via an active transport mechanism [19]. Another recent approach employs a longer hydrophobic chain heptyl ester derivative of zanamivir with ion-pairing by 1-hydroxy-2-naphthoic acid to enhance the lipophilicity and promote permeability [20]. Increased zanamivir absorption has been observed with these alternative compositions but their pursuit as development targets is complicated by regulatory requirements that would require a New Drug Application (NDA) and a full drug development program. Alternatives that can enhance absorption of the existing, unmodified approved drug, zanamivir, would have a much shorter regulatory pathway. Our results demonstrate that simple formulations composed of zanamivir in combination with generally recognized as safe (GRAS) permeability enhancers offer an ability to dramatically increase intestinal absorption and a potentially convenient route to an orally available dosage form of a variety of polar neuraminidase inhibitors.

## Materials and Methods

### Materials

Zanamivir was obtained from Beijing APIfocus Co., Beijing, China. Capmul MCM L8 was obtained from Abitec Corporation, Janesville, WI. Glycerol was obtained from Fisher Scientific, Fair Lawn, NJ. All other reagents were of the highest purity commercially available.

### Methods

#### Ethics Statement

This study was carried out in strict accordance with the recommendations in the Guide for the Care and Use of Laboratory Animals of the National Institutes of Health. The protocol was approved by the Institutional Animal Care and Use Committee of Absorption Systems, LP (Assurance Number: A4653-01). All efforts were made to minimize suffering and discomfort during the course of the study.

#### Caco-2 Cell Culture

Caco-2 cells were obtained from American Type Culture Collection (Rockville, MD). Stock cultures were maintained in flasks with DMEM medium supplemented with 10% FBS, 1% non-essential amino acids, 1 mmol/L sodium pyruvate, 100 IU/mL penicillin, and 100 µg/mL streptomycin in a humidified incubator (37°C, 5% CO_2_). Cells were harvested by trypsinization and seeded at 60,000 cells/cm^2^ onto Costar Transwell® 12-well dual-chamber plates with collagen-coated, microporous polycarbonate membranes (1.13 cm^2^ insert area, 0.4 µm pore size; Corning Life Sciences, Acton, MA) for permeability studies. The culture medium was changed three times per week.

#### Certification of Cells for the Study

Caco-2 cell monolayers that had grown for at least 20 days were subjected to batch quality control testing in which permeation rates were measured for atenolol, digoxin, estrone-3-sulfate, lucifer yellow (LY), and propranolol. In addition, the transepithelial electrical resistance (TEER) across each experimental monolayer was tested prior to an experiment.

#### Tolerability Assessment of Excipients in Caco-2 Cell Monolayers

Tolerability was assessed with zanamivir (15 µg/mL) in the presence and absence of the two excipients (each at 5%) in Caco-2 cell monolayers, based on the rate of permeation of the fluorescent monolayer integrity marker LY immediately post-exposure and after 4 hr recovery.

#### Unidirectional Permeability of Zanamivir across Caco-2 Cell Monolayers in the Presence and Absence of Excipients

Unidirectional (A-to-B) permeability of zanamivir was determined at 15 µg/mL in the absence and presence of varying concentrations of excipients in Caco-2 cell monolayers. The assay buffer was Hanks' balanced salt solution (HBSS) supplemented with 10 mM 4-(2-hydroxyethyl)-1-piperazineethanesulfonic acid (HEPES) and 15 mM D-glucose (HBSSg), pH 7.4. The dosing solutions on the apical side (0.5 mL) contained either zanamivir and excipients at each concentration being tested or zanamivir only (n = 4), while the receiver buffer in the bottom well (1.5 mL) was always excipient-free. The Caco-2 cell monolayers were incubated for 120 min in a humidified incubator (37°C, 5% CO_2_) after dosing in the apical chambers. Aliquots (200 µL each) of receiver buffer were sampled at 60 min, 90 min, and 120 min after dosing, with replacement by the same volume of blank buffer (at 60 min and 90 min). Donors were sampled at 0 and 120 min.

To assess the viability of the cells after the incubation, the cells were trypsinized and counted, after mixing with trypan blue, with an automated cell counter (Countess™, Life Technologies, Grand Island, NY), which reports total, living, and dead cell numbers, and % viability.

#### Animal Procedures

Male Sprague-Dawley rats (Hilltop Labs, Scottdale, PA), 3 animals per treatment group, 250–350 grams in weight, were fitted with a jugular vein cannula (JVC). Animals intended for intraduodenal dosing were also fitted with one or more intraduodenal cannula (IDC) and those intended for intravenous dosing were fitted with a second JVC. Food was withheld from the animals for a minimum of twelve hours prior to test article administration and was returned approximately four hours post-dose. Water was supplied *ad libitum*.

Intraduodenal doses composed of 1.5 mg of zanamivir and varying amounts of an enhancer (glycerol or Capmul MCM L8) were injected directly into the duodenum via the IDC. To determine absolute bioavailability of the intraduodenal administered drug, intravenous dosing was conducted by the injection of 1.5 mg of zanamivir in 200 µL of PBS through a JVC. For some experiments, the absorption enhancer was administered intraduodenally 2 hr prior to administering 1.5 mg of zanamivir in PBS through a second IDC. Each intraduodenal dose was followed with the introduction of a small air bubble (∼10 µL) in the cannula followed by a flush of 125 µL of PBS to ensure the dose was given in full. The volume of PBS used for cannula flush was consistent across the treatment groups. The cannula was tied to prevent the PBS remaining in the cannula from entering the duodenum.

Blood samples collected via the JVC, approximately 400 µL each, were obtained at 2 min, 5 min, 15 min, 30 min, 60 min, 90 min, and 120 min, with sodium heparin used as an anti-coagulant. Each sample was placed into a chilled tube containing the anticoagulant and kept on ice until centrifugation at 4°C, 3,000× g, for 5 min. The plasma supernatants were stored at −70°C until LC-MS analysis.

#### Analytical Method for Zanamivir Quantitation

Dosing solution samples were assayed by liquid chromatography-mass spectrometry (LC-MS/MS) using electrospray ionization. The chromatographic system consisted of Perkin Elmer series 200 micropumps and autosampler equipped with a Waters Atlantic® HILIC Silica 3 µM, 2.1×50 mm column. The mass spectrometer was a PE Sciex API 4000 with electrospray interface in multiple reaction monitoring mode. Specificity of the analytical method was evaluated and neither of the excipients was found to interfere with the analysis of zanamivir. Stock solutions (1 mg/mL zanamivir) were prepared in water. Standards (eight concentrations) were prepared in the appropriate matched matrix (HBSSg or Sprague-Dawley rat plasma containing sodium heparin) and diluted 50-fold with methanol. Experimental samples were identically treated.

## Results

### Impact of permeability enhancers on the transport of neuraminidase inhibitors across Caco-2 cell monolayers

Initial screening experiments testing transport of the neuraminidase inhibitor peramivir across Caco-2 cell monolayers, utilizing over 20 potential permeability enhancer compounds or compositions, demonstrated a broad range of impact on drug permeability (results not shown). Two enhancers, glycerol and Capmul MCM L8, provided substantially increased drug transport across Caco-2 cell monolayers and were selected for a more extensive evaluation with an alternate neuraminidase inhibitor, zanamivir, the active ingredient in the drug Relenza® (GlaxoSmithKline, Boston, MA, USA).

**Figure 1:**
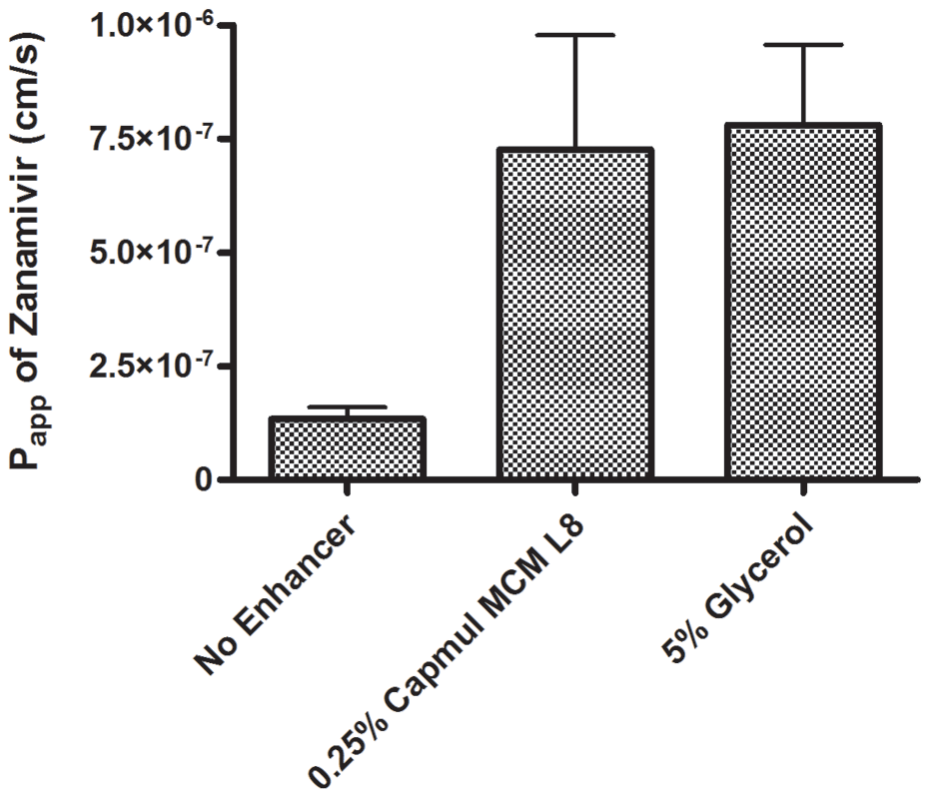
Permeability of zanamivir through Caco-2 cell monolayers in the absence and presence of absorption enhancers. The enhancers 0.25% Capmul MCM L8 and 5% glycerol resulted in 5.2- and 5.6-fold increased permeability, respectively, compared to the no-enhancer negative control. Zanamivir had significantly higher (p<0.01) permeability in the presence of the indicated enhancers compared to the PBS control. Error bars depict standard deviation.

Figure 1 shows results of Caco-2 cell assays with zanamivir utilizing optimized concentrations of both Capmul MCM L8 and glycerol. Optimized concentrations of each were derived based upon conditions found to provide the highest permeability and no impact on Caco-2 cell viability and membrane integrity (viability and tolerability test results not shown). Under the optimized conditions, both Capmul MCM L8 and glycerol provided over a 5-fold increase in the apparent permeability coefficient (P_app_) of zanamivir. Capmul MCM L8 was an inherently more potent permeability enhancer with zanamivir, as a similar increase in zanamivir transport across the membrane was observed at a 20-fold lower concentration than with glycerol. These results demonstrated a clear ability for permeability enhancers to increase the transport of zanamivir across a biological barrier (a monolayer of intestinal epithelial cells); therefore, the study was expanded to explore the potential of these enhancers for increased intestinal absorption in a rat model system.

### Impact of permeability enhancers on zanamivir absolute bioavailability in rats

Results from Caco-2 cell monolayer permeability studies suggested the permeability enhancers glycerol and Capmul MCM L8 could provide a significant increase in zanamivir absorption despite its high polarity and inherently low oral absolute bioavailability of under 2% [3], [14]. Experiments to test the ability of permeability enhancers to increase the absolute bioavailability of polar neuraminidase inhibitors containing guanidino groups indicated there was no absorption enhancement effect when delivered orally directly into the stomach (results not shown), presumably due to dilution of the enhancer. As a result, the experiments presented were designed using intraduodenal administration of the drug/enhancer formulation in an effort to mimic the performance of an enteric coated dosage form to be released in the duodenum.

**Figure 2:**
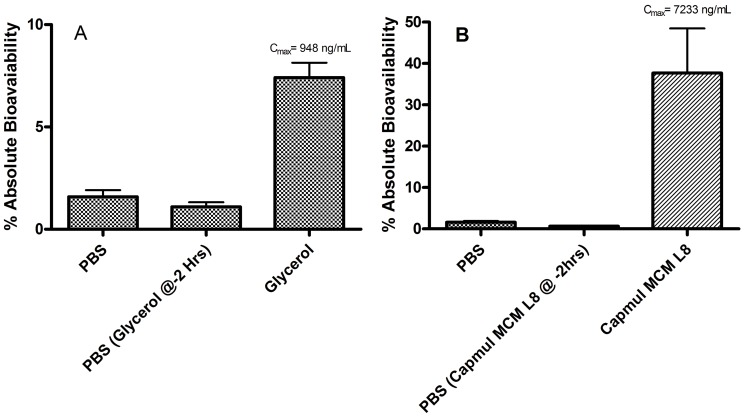
Absolute bioavailability of zanamivir after intraduodenal administration of 1.5 mg of zanamivir in 50 µL of the indicated test vehicle in rats. As indicated, in some experiments the test vehicles glycerol and Capmul MCM L8 were administered 2 hr prior to the administration of zanamivir in PBS. Panel A, Glycerol as test vehicle; Panel B, Capmul MCM L8 as test vehicle. Zanamivir had significantly higher (p<0.01) absolute bioavailability when dosed in the Capmul MCM L8 formulation compared to all other formulations. Error bars depict standard deviation.

**Figure 3:**
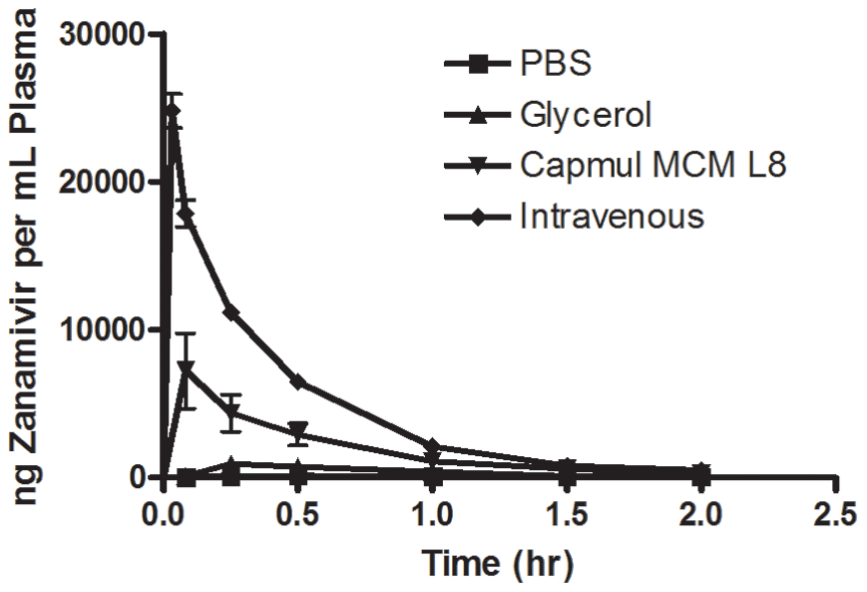
Timecourse of changes in plasma zanamivir concentrations with differing intraduodenally administered formulations versus intravenous administration of zanamivir in PBS. A rapid uptake from the intraduodenally administered Capmul MCM L8 formulation was observed which was rapidly cleared in a manner corresponding to the intravenous administration route. All animals were dosed with 1.5 mg of zanamivir regardless of dosing route or formulation. Error bars depict standard deviation.

Figure 2 depicts results from studies using intraduodenal administration of zanamivir/enhancer formulations in male Sprague-Dawley rats. In these experiments, rats fitted with a cannula in the duodenum were administered 1.5 mg of zanamivir in 50 µL vehicles composed of either PBS, glycerol, or Capmul MCM L8. The results demonstrate low absorption of zanamivir in the absence of enhancer, along with dramatically increased absolute bioavailability in their presence. The absolute bioavailability of zanamivir was increased 4.7- and 23.7-fold in 50 µL of glycerol and Capmul MCM L8, respectively, compared to PBS. In Table 1, the pharmacokinetic parameters for zanamivir using the indicated formulations are presented. Most notably, a C_max_ of over 7000 ng/mL was achieved when Capmul MCM L8 was used as the enhancer. Figure 3 depicts the plasma zanamivir concentrations over time. These results demonstrate a rapid intraduodenal uptake of the drug and its clearance.

**Table 1:** Summary of Pharmacokinetic Parameters for Zanamivir from Different Formulations after Intraduodenal Administration in Male Sprague-Dawley Rats at 1.5 mg/animal.

*PK Parameter*	PBS	Glycerol	Capmul MCM L8	PBS (Glycerol @ -2 hr)	PBS (Capmul MCM L8 @ -2 hr)
**C_max_ (ng/mL)**	134	948	7233	77.4	48.6
**t_max_ (hr)**	0.5	0.5	0.08	0.58	1
**t_1/2_ (hr)**	0.83	0.29	0.49	1.24	0.86
**AUC_last_ (hr·ng/mL)**	162	754	3803	112	66.1
**AUC_∞_ (hr·ng/mL)**	244	781	4005	156	69.8
***Dose-normalized Values*** 1					
**AUC_last_ (hr·kg·ng/mL/mg)**	30.6	145	715	21.1	12.7
**AUC_∞_ (hr·kg·ng/mL/mg)**	46.6	153	753	29.1	13
**Bioavailability (%)**	1.59	7.53	37.7	1.1	0.66

C_max_: Maximum plasma concentration; t_max_: Time to maximum plasma concentration; t_1/2_: half-life; AUC_last_: Area Under the Curve, calculated to the last observable time point; AUC_∞_: Area Under the Curve, extrapolated to infinity;

1 Dose normalized by dividing the parameter by the nominal dose of 1.5 mg/animal.

As an initial test of the duration of the permeability enhancement effect of glycerol and Capmul MCM L8, experiments were conducted in which the permeability enhancers were administered 2 hr prior to zanamivir dosing (see Figure 2A and 2B). In these experiments, temporal separation of the enhancer and drug by 2 hr resulted in no enhanced absorption; for both enhancers, the absolute bioavailability was equivalent to that of the negative control. Clearly, the enhancement effect is transient and lasts well under 2 hr.

### Variation of intraduodenal enhancer and zanamivir levels on absolute bioavailability

**Figure 4:**
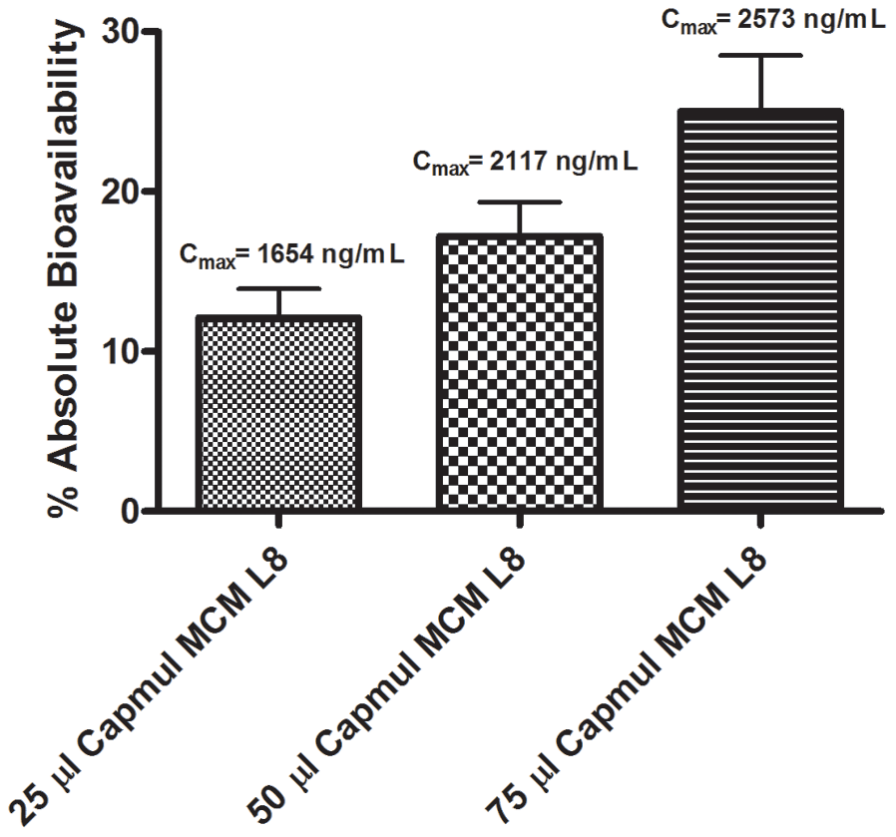
Effect of increasing Capmul MCM L8 on the absolute bioavailability of 1.5 mg of zanamivir after intraduodenal administration in rats. Zanamivir had significantly higher (p<0.01) absolute bioavailability when dosed in the Capmul MCM L8 formulation compared to the control and there was a significant difference (p<0.05) in the absolute bioavailability observed between 25 µL and 75 µL Capmul MCM L8 volume dosed groups. Error bars depict standard deviation.

The effect of increasing intraduodenally administered Capmul MCM L8 at a fixed 1.5 mg zanamivir drug load on absolute bioavailability is shown in Figure 4. A roughly linear increase in both absolute bioavailability of zanamivir and C_max_ are observed as Capmul MCM L8 amounts are increased 3-fold from 25 µL to 75 µL. These results demonstrate that enhancer amounts can be varied to optimize drug absorption and the associated pharmacokinetic parameters.

**Figure 5:**
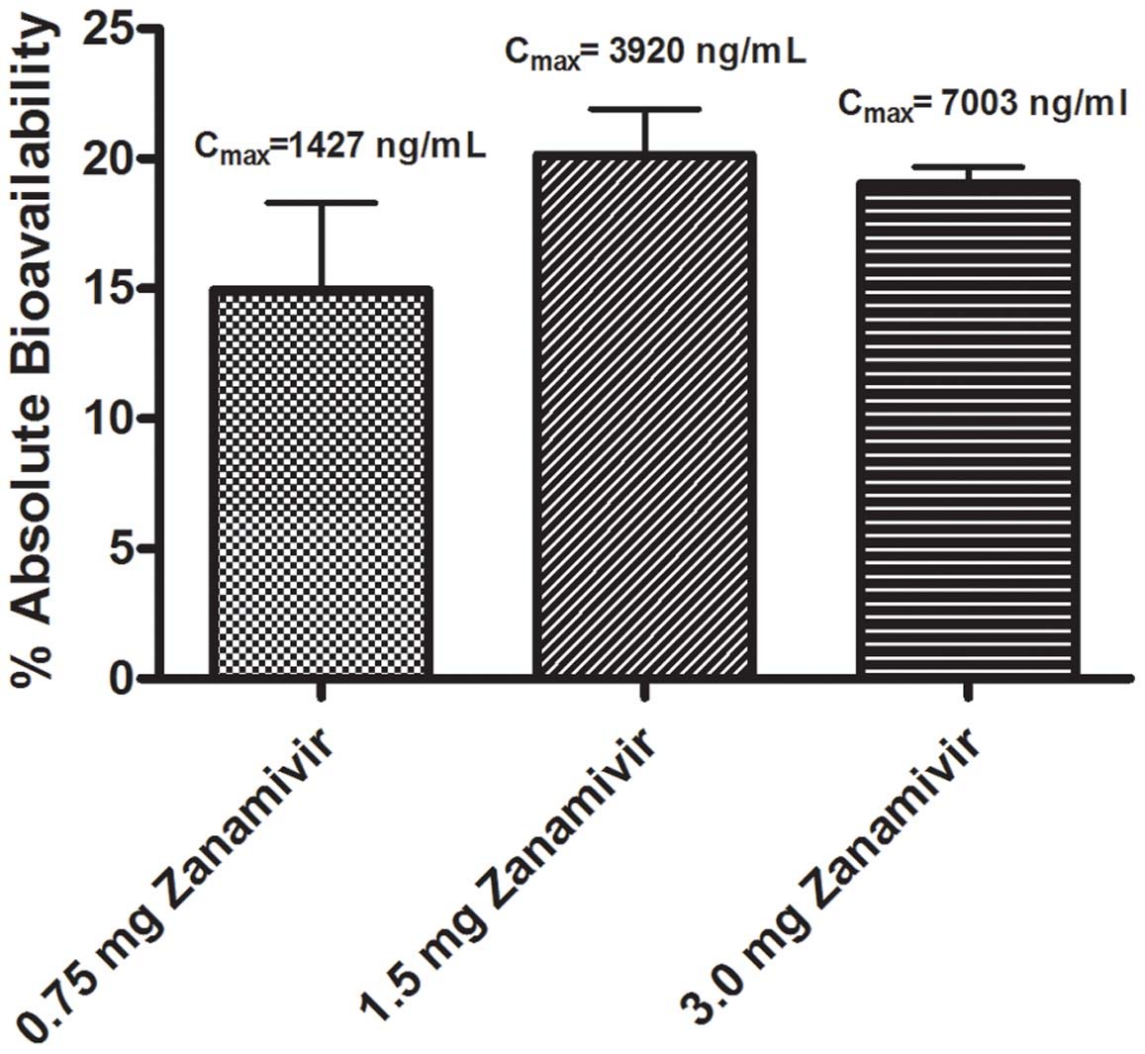
Effect of increasing zanamivir in the presence of 50 µL of Capmul MCM L8 on zanamivir absolute bioavailability after intraduodenal administration in rats. Zanamivir had significantly higher (p<0.01) absolute bioavailability when dosed in the Capmul MCM L8 formulation compared to the PBS control. Error bars depict standard deviation.

Figure 5 summarizes the reciprocal results from varying zanamivir levels at a fixed 50 µL amount of Capmul MCM L8 after intraduodenal administration. Although there was only a modest difference in the absolute bioavailability of zanamivir as the dose varied 4-fold from 0.75 mg to 3.0 mg, there was a substantial impact on the resulting C_max_. The C_max_ varied roughly proportionately to the drug load, with a short t_max_ of 0.08, 0.08, and 0.14 hr for the 0.75 mg, 1.5 mg, and 3.0 mg zanamivir dosages, respectively. These results suggest that once the enhancer opened tight junctions to facilitate paracellular absorption, very rapid drug uptake occurred for a short duration, presumably due to only transient stimulation of the paracellular pathway.

## Discussion

Viral neuraminidase inhibitors (oseltamivir, zanamivir, laninamivir, peramivir) bind to influenza virus neuraminidase with high specificity and affinity and have been used therapeutically for the treatment of acute influenza [3]. In contrast to influenza vaccines, neuraminidase inhibitors are not intended to prevent influenza infection, but have the advantage of lacking much of the strain specificity of vaccines, thereby providing more generalized utility in treatment to lessen the symptoms and shorten their duration. The broad utility of neuraminidase inhibitors among influenza virus strains is associated with the conserved sequence within the neuraminidase active site [5]. However, associated with the therapeutic use of neuraminidase inhibitors, in particular Tamiflu® (oselatamivir), has been the appearance of resistant strains, which lessens the value of a single therapeutic compound. For example, 2009 tests of seasonal H1N1 and pandemic flu strains were 99.6% and 0.5% resistant to Tamiflu®, respectively [21]. In contrast, zanamivir resistance has not been observed [21] and the reported Tamiflu® resistant flu strains remain zanamivir sensitive. Thus, at present and until resistant strains to other neuraminidase inhibitors appear and limit their utility, zanamivir and other neuraminidase inhibitor compounds become important alternate drugs for influenza treatment.

Neuraminidase inhibitors were obtained through rational drug design as transition state analogs to achieve high enzyme specificity and affinity. Therapeutic effects are caused by saturating the enzyme with inhibitor and reliance on their high affinity and slow off-rate of binding to provide a sustained impact on viral replication between dosing intervals [3]. For oral or intravenous formulations, this involves achievement of a high C_max_ capable of saturating the enzyme consistent with Michaelis-Menten enzyme kinetics. In the case of inhaled formulations, high local concentrations of inhibitor are achieved at the surface of infected cells, resulting in the same high level of inhibitor binding to the enzyme.

Inherent to their molecular design is the presence of key polar functional groups, including a negatively charged carboxylic acid group and a positively charged primary amine or guanidino group. These functional groups give rise to the overall high polarity of this class of drugs, and their failure to be recognized by active transporter proteins renders them Biopharmaceutical Classification System (BCS) class 3 drugs (high solubility, low permeability). As a result, drug delivery is a challenge.

Efforts to address high polarity with a resulting increase in intestinal permeability were successful in the case of oseltamivir carboxylate, which contains both a carboxyl group and a primary amine [17]. The absolute bioavailability of oseltamivir carboxylate was increased from under 5% to 35% or greater by blocking the carboxylic acid group with an ethyl ester. Once absorbed, esterases cleave the ester linkage, yielding the active oseltamivir carboxylate drug form. In contrast, a simple ethyl ester prodrug approach was not capable of enhancing the intestinal absorption of a variant of oseltamivir carboxylate, GS4116 carboxylate, containing instead the much more basic guanidino group. GS4116 carboxylate is a 10-fold more potent inhibitor than oseltamivir carboxylate, with oral bioavailability under 5% [16]. The GL4116 ethyl ester derivative, despite being somewhat less polar, retains a bioavailability of under 5%. Thus, despite the 10-fold increased potency of the guanidino-containing variant, bioavailability limitations were a major driver in the design of the marketed product. The simple ester modification of oseltamivir carboxylate, creating a prodrug structure, has allowed Tamiflu® to be delivered orally. This convenient dosing route has allowed Tamiflu® to dominate the market. The pattern of very poor oral bioavailability among neuraminidase inhibitors containing a guanidino group (zanamivir, laninamivir, and peramivir) is consistent. Each has oral bioavailability under about 2%. This has required the use of alternative drug delivery strategies such as inhalation or intravenous formulations [3], [14].

Taken together, the results of this study demonstrate enhanced absorption of the very polar neuraminidase inhibitor zanamivir when delivered intraduodenally in rats. Significantly increased absolute bioavailability at a high C_max_ was observed with some variation in the degree of absolute bioavailability increase observed between experiments due to common variability between animals and experiments. The results demonstrated that variation of both enhancer and drug amounts in a formulation can be used to achieve a desired absolute bioavailability and C_max_ for optimization of human formulations for oral zanamivir administration. The short t_max_ and transient stimulation of the paracellular pathway associated with zanamivir absorption suggests that oral formulations for human use in influenza treatment should be in enteric-coated dosage forms to allow rapid release in the duodenum at relatively high enhancer and zanamivir local concentrations. The results presented provide a strong basis for their extension into the creation of an oral zanamivir formulation for influenza treatment.

## Conclusions

The results presented demonstrate that absorption enhancers, when present in high concentration in the duodenum, give rise to a dramatic increase in the absolute bioavailability and high C_max_ of very polar neuraminidase inhibitors. This provides a new and simple potential formulation alternative for the oral administration of polar neuraminidase inhibitors comprising drug and absorption enhancer in an enteric coated dosage form for influenza treatment in humans.

## References

[1] World Health Organization (2009) World Health Organization Fact Sheet 211. April 2009

[2] Nicholls H (2006) Pandemic Influenza: The Inside Story. PLoS Biology 4: e50. 16464130 10.1371/journal.pbio.0040050 PMC1363710

[3] Moscona A (2005) Neuraminidase inhibitors for influenza. N Engl J Med 353: 1363–1373. 16192481 10.1056/NEJMra050740

[4] Garman E, Laver WG (2005) The structure, function, and inhibition of influenza virus neuraminidase. *In*: Viral Membrane Proteins: Structure, Function, and Drug Design, Wolfgang Fischer (ed). Kluwer Academic/Plenum Publishers, New York.

[5] Coleman PM (1994) Influenza viral neuraminidase: structure, antibodies, and inhibitors. Protein Science 3: 1687–1696. 7849585 10.1002/pro.5560031007 PMC2142611

[6] Vagrhese JN, Smith PW, Sollis SL, Blick TJ, Sahasrabudhe A, et al (1998) Drug design against a shifting target: a structural basis for resistance to inhibitors in a variant of influenza virus neuraminidase. Structure 6: 735–746. 9655825 10.1016/s0969-2126(98)00075-6

[7] Kim CU, Lew W, Williams MA, Liu H, Zhang L, et al (1997) Influenza neuraminidase inhibitors possessing a novel hydrophobic interaction in the enzyme active site: design, synthesis, and structural analysis of carbocyclic sialic acid analogues with potent anti-influenza activity. J Am Chem Soc 119: 681–690. 16526129 10.1021/ja963036t

[8] Russell RJ, Haire LF, Stevens DJ, Collins PJ, Pin YP, et al (2006) The structure of H5N1 avian influenza neuraminidase suggests new opportunities for drug design. Nature 443: 445–49. 10.1038/nature05114 16915235

[9] Babu YS, Chand P, Bantia S, Kotian P, Dehghani A, et al (2000) BCX-1812 (RWJ-270201): discovery of a novel, highly potent, orally active, and selective influenza neuraminidase inhibitor through structure-based drug design. J Med Chem 43: 3482–6. 11000002 10.1021/jm0002679

[10] Sudbeck EA, Jedrzejas MJ, Singh S, Brouillette WJ, Air GM, et al (1997) Guanidinobenzoic acid inhibitors of influenza virus neuraminidase. J Mol Biol 267: 584–94. 9126840 10.1006/jmbi.1996.0885

[11] Chand P, Babu YS, Bantia S, Chu N, Cole LB, et al (1997) Design and synthesis of benzoic acid derivatives as influenza neuraminidase inhibitors using structure-based drug design. J Med Chem 40: 4030–52. 9406595 10.1021/jm970479e

[12] von Itzstein M, Wu WY, Kok GB, Pegg MS, Dyason JC, et al (1993) Rational design of potent sialidase-based inhibitors of influenza virus replication. Nature 363: 418–23. 8502295 10.1038/363418a0

[13] Aoki FY, Hayden FG (1999) Zanamivir. A potent and selective inhibitor of influenza A and B viruses. Clin Pharmacokinet 36 Suppl 1: v–ix. 10429834

[14] Cass LM, Efthymiopoulos C, Bye A (1999) Pharmacokinetics of zanamivir after intravenous, oral, inhaled or intranasal administration to healthy volunteers. Clin Pharmacokinet 36 Suppl 1: 1–11. 10.2165/00003088-199936001-00001 10429835

[15] García-Sosa AT, Sild S, Maran U (2008) Design of multi-binding-site inhibitors, ligand efficiency, and consensus screening of avian influenza H5N1 wild-type neuraminidase and of the oseltamivir-resistant H274Y variant. J Chem Inf Model 48: 2074–2080. 18847186 10.1021/ci800242z

[16] Li W, Escarpe PA, Eisenberg EJ, Cundy KC, Sweet C, et al (1998) Identification of GS 4104 as an orally bioavailable prodrug of the influenza virus neuraminidase inhibitor GS 4071. Antimicrob Agents Chemother 42: 647–53. 9517946 10.1128/aac.42.3.647 PMC105512

[17] Mendel DB, Tai CY, Escarpe PA, Li W, Sidwell RW, et al (1998) Oral administration of a prodrug of the influenza virus neuraminidase inhibitor GS 4071 protects mice and ferrets against influenza infection. Antimicrob Agents Chemother 42: 640–6. 9517945 10.1128/aac.42.3.640 PMC105511

[18] Miller JM, Dahan A, Gupta D, Varghese S, Amidon GL (2010) Enabling the intestinal absorption of highly polar antiviral agents: ion-pair facilitated membrane permeation of zanamivir heptyl ester and guanidino oseltamivir. Mol Pharm 7: 1223–34. 20536260 10.1021/mp100050d PMC3496398

[19] Gupta SV, Gupta D, Sun J, Dahan A, Tsume Y, et al (2011) Enhancing the intestinal membrane permeability of zanamivir: a carrier mediated prodrug approach. Mol Pharm. 8: 2358–67. 21905667 10.1021/mp200291x PMC3304100

[20] Liu KC, Lee PS, Wang SY, Cheng YS, Fang JM, et al (2011) Intramolecular ion-pair prodrugs of zanamivir and guanidine-oseltamivir. Bioorg Med Chem 19: 4796–802. 21778065 10.1016/j.bmc.2011.06.080

[21] 2008–2009 Influenza Season Week 32 ending August 15, 2009. Flu Activity & Surveillance. Centers for Disease Control and Prevention (CDC). August 21, 2009. Available: http://www.cdc.gov/flu/weekly/weeklyarchives2008-2009/weekly32.htm

